# Contrast-enhanced CT in sepsis: insights from a European Emergency Radiology survey

**DOI:** 10.1007/s00330-025-12256-y

**Published:** 2026-01-26

**Authors:** Ann-Christine Stahl, Kerstin Rubarth, Maria Isabel Opper Hernando, Anne Frisch, Ana Blanco-Barrio, Raffaella Basilico, Vittorio Miele, Francesca Iacobellis, Myrto Bolanaki, Denis Witham, Marc Dewey, Julian Pohlan

**Affiliations:** 1 Department of Radiology, University of Cambridge, Cambridge University Hospitals, Cambridge, UK; 2 Department of Radiology, Charité—Universitätsmedizin Berlin, Corporate Member of Freie Universität Berlin and Humboldt-Universität zu Berlin, Berlin, Germany; 3 Institute of Biometry and Clinical Epidemiology, Charité—Universitätsmedizin Berlin, Corporate Member of Freie Universität Berlin and Humboldt-Universität zu Berlin, Berlin, Germany; 4 Emergency Section, Department of Radiology, Hospital Universitario Morales Meseguer, Murcia/ES, Spain; 5 Department of Medical, Oral and Biotechnological Sciences, University G.D’Annunzio Chieti-Pescara, Via dei Vestini, Chieti, Italy; 6 Department of Biomedical Experimental and Clinical Sciences, University of Florence, Florence, Italy; 7 Department of Radiology, Careggi University Hospital, Florence, Italy; 8 Department of General and Emergency Radiology, “A. Cardarelli” Hospital, Naples/IT, Italy; 9 Emergency Department, Charité—Universitätsmedizin Berlin, Freie Universität Berlin and Humboldt-Universität zu Berlin, Berlin, Germany; 10 Innere Medizin—Kardiologie, Allgemeine Innere Medizin und konservative Intensivmedizin, Vivantes Klinikum Am Urban, Berlin, Germany; 11 Berlin Institute of Health at Charité—Universitätsmedizin Berlin, Berlin, Germany; 12 Johnson & Johnson Innovative Medicine, Janssen-Cilag GmbH, Neuss, Germany

**Keywords:** Sepsis, Tomography (X-ray computed), Emergencies, Contrast media

## Abstract

**Objectives:**

To gain insight into emergency radiologists’ views on the role of contrast-enhanced CT (CECT) in sepsis management.

**Materials and methods:**

This analysis of a survey distributed in 2023 to members of the European Society of Emergency Radiology (ESER) (*n* = 297) gathered perspectives on the role of CECT in patients with sepsis. The previously validated questionnaire used for this survey encompassed demographic information, clinical experience, and inquiries regarding the timing and rationale for CT. Results were compared with data from a prior single-center survey among clinicians and general radiologists. Responses were collected anonymously and analyzed using descriptive statistics and chi-square tests. As not all items were mandatory, item-specific response numbers may vary and are reported accordingly throughout the manuscript.

**Results:**

A total of 144 emergency radiologists participated, with most of them endorsing a 1–6-h timeframe for CECT after the diagnosis of sepsis (45.8%; *n* = 27/59). However, a notable proportion accepted longer intervals > 12 h (35.6%; *n* = 21/59). Emergency radiologists particularly opted for repeat imaging in patients with sepsis and clinical deterioration (35.3%; 24/68), whereas clinicians tended to be more hesitant (2.9%; *n* = 10/341).

**Conclusion:**

The results of this survey indicate strong agreement among emergency radiologists on the relevance of prompt CECT for the timely diagnostic management of patients with sepsis. Several aspects related to timing and indication show significant interdisciplinary differences, requiring further study.

**Key Points:**

***Question*** *While contrast-enhanced CT can aid in detecting the infectious focus, this study explores emergency radiologists’ perspectives on its role in sepsis management*.

***Findings*** *Radiologists and clinicians from different subspecialties agree on the importance of promptly performed (≥ 1–6 h) CECT in sepsis management and the contraindications of contrast application*.

***Clinical relevance*** *This survey shows that radiologists and clinicians largely agree on the importance of CECT in sepsis management*.

**Graphical Abstract:**

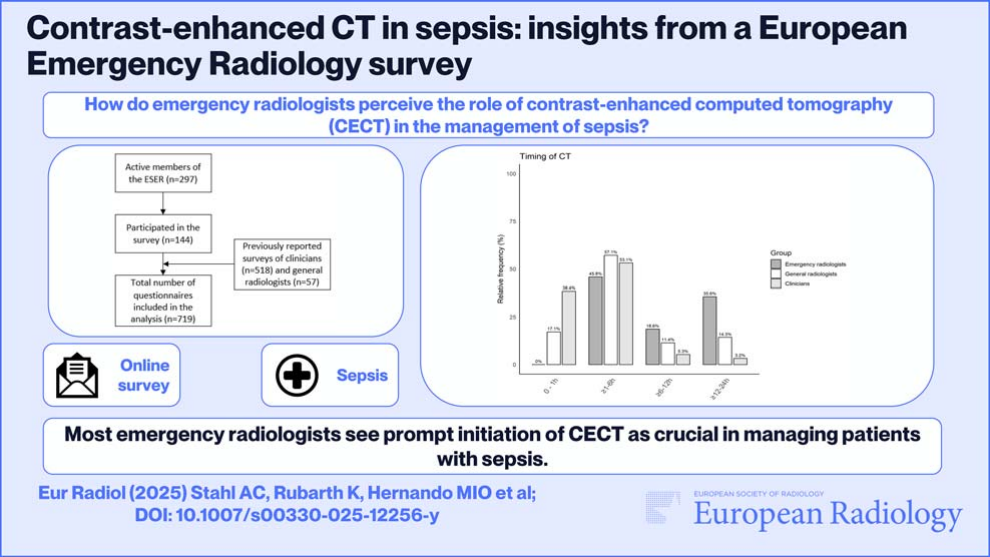

## Introduction

The role of contrast-enhanced CT (CECT) in the diagnostic pathway of patients with sepsis remains insufficiently defined in current clinical guidelines, despite its frequent use in practice [1–3]. While recommendations consistently emphasize the need for early identification of the infectious focus and initiation of appropriate antibiotic therapy, they fail to specify the contribution of CECT imaging to this process [4–7]. Critical questions regarding optimal timing, indications, and contraindications for the use of CECT in patients with sepsis remain unanswered.

The diagnosis of sepsis relies on clinical evaluation and laboratory parameters, with limited diagnostic accuracy of screening tools [4, 8]; the clinical assessment, especially, may fall short in individuals unable to cooperate due to complications such as septic encephalopathy [5, 9, 10]. While previous guidelines suggested imaging techniques for focus identification, current recommendations lack specific directives on when and how to apply CECT in patients with sepsis [4, 5, 7].

Nonetheless, evidence indicates that CECT plays a vital role in confirming suspected infectious foci, which in turn guides further choice of antibiotic regimen, surgical intervention, or other interventional procedures [2, 11–15]. The impact of CECT on clinical decision-making is especially pronounced in emergency settings, yet systematic scientific data regarding its efficacy in diagnosing septic foci remain limited.

We previously analyzed final-year medical students’ knowledge of CECT in patients with sepsis to understand potential deficits in current medical curricula [16]. Further, we surveyed physicians’ perspectives on CECT in sepsis among different clinical specialties, including surgery and internal medicine, in comparison to radiologists’ perspectives [17]. While a specialized protocol for imaging critically ill patients is increasingly available in many institutions, we noticed a lack of data reflecting emergency radiologists’ perspectives in the diagnostic work-up of sepsis [11, 12].

Therefore, the objective of the present study is to explore the perspective of emergency radiologists on the use of CECT for patients with sepsis, focusing on the timing of CECT, indications for its utilization, and contraindications. To this end, we conducted a structured survey distributed among members of the European Society of Emergency Radiology (ESER).

## Materials and methods

### Study design

The local ethics committee approved the study (reference number EA1/203/21). The study was conducted in accordance with the Declaration of Helsinki. The study is reported in accordance with the “Strengthening the Reporting of Observational Studies in Epidemiology” (STROBE) statement for cohort studies [18–20].

The ESER study was performed prospectively with the statistical analysis plan established post hoc. In this study, all members of the ESER were contacted and asked to participate in an online survey between January 2nd, 2023, and May 3rd, 2023. The study cohort was chosen to reflect the perspectives of emergency radiologists, as ESER membership indicates a clear professional focus and special interest in emergency imaging, and only registered members received the survey invitation. Data from a previously published study [17, 21] were included in this analysis to compare the views of emergency radiologists from the ESER survey with those of general radiologists (radiologists without a defined subspecialty focus, such as emergency radiology) and clinicians (physicians from clinical specialties such as surgery, internal medicine, or anesthesiology). An email containing the link to a digital survey was sent to all members of the ESER, with no exclusion criteria applied. Previously reported surveys of clinicians from different specialties and general radiologists were included in the final analysis as explained above. Only single-time participation was permitted; participation from physicians of the same hospital was allowed.

### Questionnaire structure

An interdisciplinary team (M.I.O.H., M.D., and J.P.) developed a questionnaire focusing on the role of CECT in patients with sepsis (electronic supplementary questionnaire). The initial version of the questionnaire was pretested with final-year medical students to assess clarity and comprehensibility [16, 17, 21]. Based on their feedback, revisions were made to improve the wording. The revised questionnaire was then applied in a single-center survey among physicians at a large European university medical center, which served as a further validation step [16, 17, 21]. The structure of the questionnaire remained unchanged, with only minimal changes in questions regarding demographics and the timing of CECT [16, 17, 21]. This stepwise approach ensured the questionnaire’s validity, applicability, and relevance for a multi-center specialized radiology cohort. The questionnaire was divided into several sections. First, physicians were required to agree to the privacy statement and indicate previous participation in the survey. Demographic data included position (resident, attending (board-certified), senior, or chief), years of work experience since the beginning of residency (specialty training), medical specialty, any sepsis-related fellowships, and their primary place of employment. Residents were classified as licensed physicians in residency training for board certification. Participants also indicated their experience in managing patients with sepsis. Responses were marked on a 4-point Likert scale, i.e., (1) strongly disagree, (2) somewhat disagree, (3) somewhat agree, (4) strongly agree, to assess perceptions of the major benefits of CECT for patients with sepsis. Another set of questions asked whether physicians agreed or disagreed with various clinical scenarios or criteria pertinent to CECT examinations. Additionally, they prioritized organ regions that should be scanned to identify foci in patients with sepsis. Furthermore, they had to state their preferred time window for CECT examinations, choosing from four options: (1) < 1 h, (2) ≥ 1–6 h, (3) ≥ 6–12 h, (4) ≥ 12–24 h. In the present analysis, we intentionally focused on questionnaire items addressing the timing, indications, and contraindications of CECT, as these aspects align with the overarching theme of diagnostic decision-making in sepsis.

### Data analysis

Data was collected anonymously using an online survey (LimeSurvey, version 5.3.25, 2022; LimeSurvey GmbH). As the data were analyzed exploratively, no sample size calculation was performed. Variables analyzed included baseline data, e.g., work experience, workplace, and medical specialty, as well as variables pertaining to the benefits and risk factors of CECT. In contrast to the methodological approach in our previous publications [16, 17, 21], we included all returned questionnaires independent of the number of items answered. Therefore, the quantity (= *n*) of the individual items may differ, but was always reported to increase transparency. This decision was made to preserve the breadth of perspectives captured and allow readers to judge the robustness of each item´s result separately while increasing comparability with the previous survey results. Descriptive statistics and exact Fisher tests were performed using R (version 4.4.1) by one reviewer (K.R., statistical guarantor of the study). The significance level was set at α < 0.05. Data were analyzed exploratively; hence, no adjustment for multiple testing was conducted. Thus, results are interpreted as hypothesis-generating.

## Results

### Study population

**Fig. 1:**
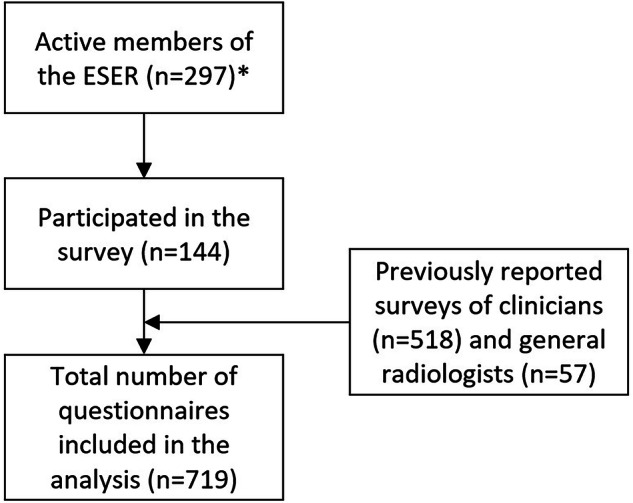
Flow-chart of survey participation. ^*^Number provided by the ESER office

In the survey presented here, we analyzed a total sample of 719 participants: 144 emergency radiologists participating in the current survey, as well as 518 clinicians and 57 general radiologists participating in an earlier survey, as described above. The flow of participants is presented in Fig. 1. More than half of the emergency radiologists (*n* = 144) were board-certified physicians, i.e., in specialized or attending positions (52.7%). More than 70% of the emergency radiologists had more than 11 years of work experience. None of the emergency radiologists were residents with less than three years of professional experience. In the earlier survey conducted in a large European university medical center [17, 21], the majority of general radiologists and clinicians were residents (50.9% and 54.5%, respectively). The majority of general radiologists and clinicians reported 3–7 years of work experience at the time of the survey. Characteristics of the participants are summarized in Table 1.

**Table 1:** Characteristics of the study population

	Clinicians	Emergency radiologists	General radiologists
*n*	518	144	57
Professional experience in radiology (%)			
Resident (radiologist in training)	256 (54.5)	3 (2.7)	29 (50.9)
Board-certified (specialist/attending) physician	110 (23.4)	59 (52.7)	13 (22.8)
Senior or chief physician	104 (22.1)	50 (44.6)	15 (26.3)
Professional experience (%)			
< 3 years	111 (23.6)	0 (0.0)	10 (17.5)
> 3 to ≤ 7 years	153 (32.6)	16 (14.8)	24 (42.1)
> 7 to ≤ 11 years	82 (17.4)	15 (13.9)	9 (15.8)
> 11 to ≤ 20 years	79 (16.8)	39 (36.1)	7 (12.3)
> 20 years	45 (9.6)	38 (35.2)	7 (12.3)
Involved in sepsis treatment = yes (%)	412 (87.5)	91 (81.2)	51 (89.5)

### Timing of CECT in sepsis

**Fig. 2:**
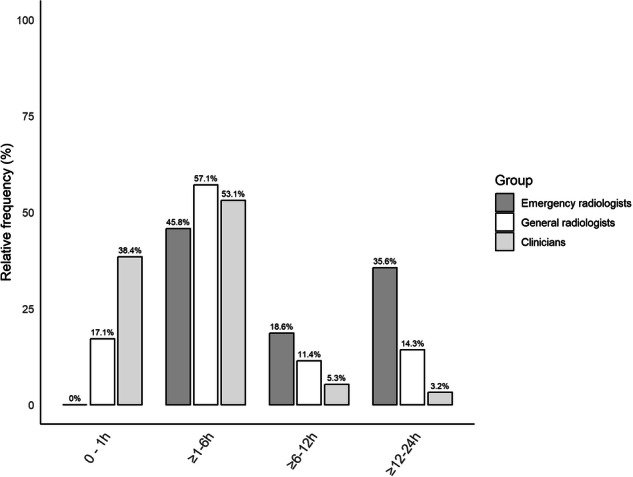
Perspectives on the timing of CECT in sepsis

**Fig. 3:**
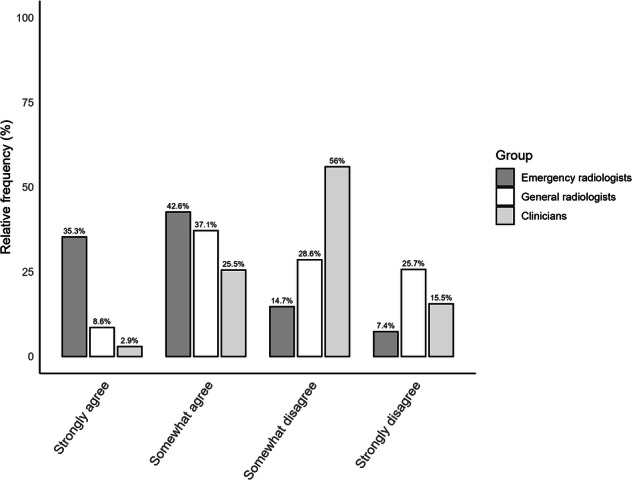
Perspectives on repeat CECT for patients with sepsis with clinical deterioration

Consistent with the results of the survey conducted among clinicians and general radiologists, the majority of specialized emergency radiologists from the ESER preferred a 1–6 h time window for diagnostic CECT in patients with sepsis (45.8–57.1% across all groups). Still, a relatively high number of emergency radiologists seemed to accept later windows > 12 h (35.6%; *n* = 21/59) for timing a scan compared with general radiologists (14.3%; *n* = 5/35) or clinicians (3.2%; *n* = 11/341). No emergency radiologist opted for a < 1 h time window (Fig. 2). Compared to general radiologists (8.6%; *n* = 3/35) or clinicians (2.9%; *n* = 10/341), emergency radiologists more often chose strongly to agree with the notion that “CT should be repeated in patients with sepsis who experience clinical deterioration” (35.3%; 24/68). Analogously, only a negligible number of emergency radiologists strongly disagreed with this notion (Fig. 3). Of note, emergency radiologists differed in professional experience from general radiologists and clinicians (Supplementary Figs. 1 and 2). Therefore, we performed a stratified analysis according to both years of professional experience and board-certification (Supplementary Fig. 3a, b). The results of this analysis indicate differences in several subgroups but do not show substantial bias.

### Perspectives on contraindications for CECT in sepsis

**Fig. 4:**
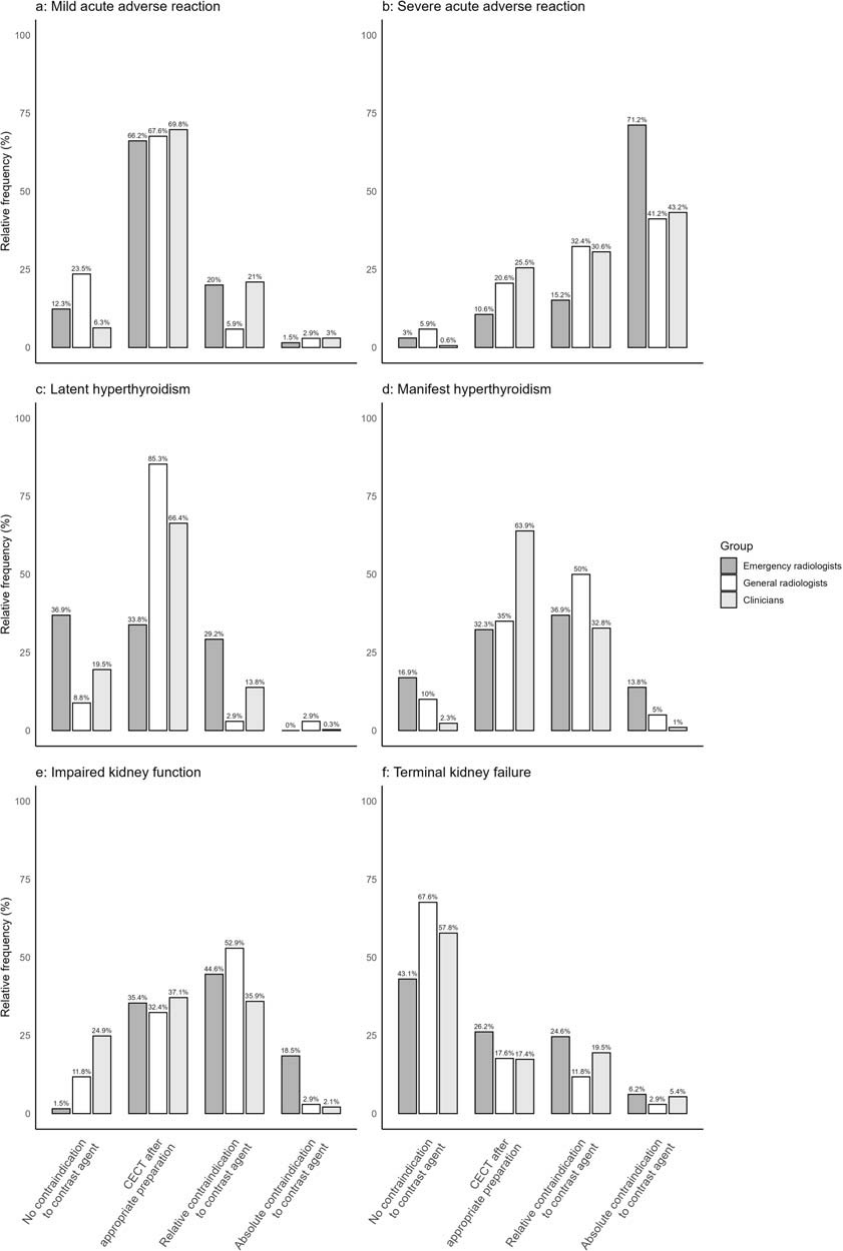
Perspectives on the role of prior mild (**a**) and severe (**b**) acute adverse reactions, of latent (**c**) and manifest (**d**) hyperthyroidism, as well as of impaired kidney function (**e**) and terminal kidney failure (**f**) in patients with sepsis as contraindications for CECT

**Fig. 5:**
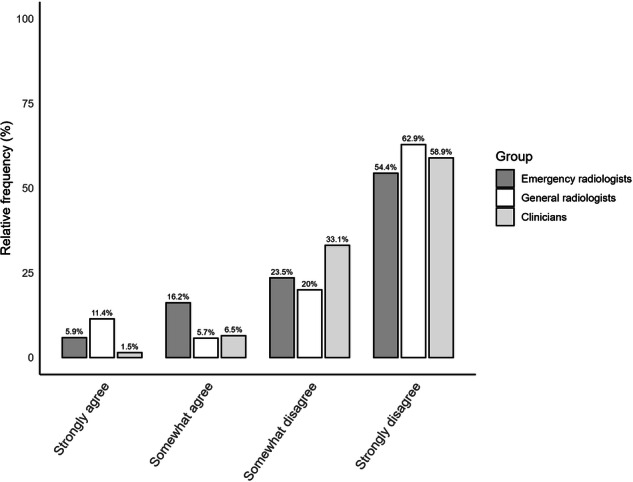
Perspectives on radiation exposure in patients with sepsis

Most participants agreed that it was possible to perform “CECT after appropriate preparation” in patients with prior mild acute adverse reactions. Still, emergency radiologists were stricter in patients with prior severe acute adverse reactions (71.2% (*n* = 47/66) in favor of absolute contraindication), in comparison to general radiologists and clinicians (41.2% (*n* = 14/34) and 43.2% (*n* = 144/333), respectively) (Fig. 4a, b). General radiologists and clinicians alike tended to advocate “CECT after appropriate preparation” in patients with latent hyperthyroidism (85.3% (*n* = 29/34) and 66.4% (*n* = 221/333), respectively), whereas emergency radiologists’ choice was “No contraindication” and “CECT after appropriate preparation”, at 70.7% in total (*n* = 46/65) (Fig. 4c). In patients with manifest hyperthyroidism, clinicians favored “CECT after appropriate preparation” (63.9%; *n* = 193/302), though 50% (*n* = 10/20) of general radiologists, as well as a small majority of emergency radiologists (36.9%; *n* = 24/65) favored “Relative contraindication” (Fig. 4d). A higher proportion of emergency radiologists saw a relative or absolute contraindication for CECT in patients with impaired kidney function (44.6% (*n* = 29/65) relative and 18.5% (*n* = 12/65) absolute contraindication), compared with clinicians and general radiologists (Fig. 4e). There is wide variation in responses regarding the use of CECT in patients with end-stage kidney disease requiring dialysis (43.1–67.6% across all groups), though the importance of appropriate preparation, e.g., hydration, was emphasized by some of the participants (17.4–26.2% across all groups, Fig. 4f). Most emergency radiologists, general radiologists, and clinicians strongly disagreed with the notion that radiation should be seen as a relevant contraindication in patients with sepsis (Fig. 5). Still, more emergency radiologists than clinicians agreed with this, i.e., 22.1% (*n* = 15/68) vs 8% (*n* = 57/341).

## Discussion

This survey provides a comprehensive analysis of emergency radiologists’ perspectives on CECT in patients with sepsis. There was overall agreement that CECT should be performed within a short time window in patients with sepsis. Still, a relevant share of emergency radiologists accepted a longer time window, while clinicians clearly tended towards a shorter time window. Furthermore, emergency radiologists demonstrated a greater inclination towards repeat imaging in patients with clinical deterioration than general radiologists and clinicians. Interestingly, clinicians tended to be more hesitant about repeated imaging. There was interdisciplinary agreement on contraindications. However, emergency radiologists were notably amenable to CECT, even for patients with latent hyperthyroidism. Besides, most emergency radiologists firmly objected to CECT in patients with a history of severe acute adverse reactions.

The specific case scenarios and their respective answers in this survey may not be covered by the general recommendations of the current guidelines, which could result in answers that differ from the guidelines. Current guidelines lack specific recommendations for the timing of CECT in patients with sepsis, and no solid database exists [1]. We previously published the results of our survey in a large European university medical center that was also used as a reference population for the current analysis [17, 21]. The participation of a sufficiently high number of emergency radiologists in this survey enabled the comparison of the results with data from the previous survey among general radiologists and clinicians. Taken together, these data clearly demonstrate that a significant number of survey participants regard the 1–6-h interval as the optimal timing for CECT in patients with sepsis. A potential rationale for the increased acceptance of extended CT time windows among emergency radiologists may be their necessity to balance multiple acute indications, such as vascular emergencies or trauma cases, which can temporarily take precedence over sepsis imaging. Conversely, clinicians are frequently guided more directly by the concept of the “golden hour” in sepsis treatment, which may result in the transfer of this urgency to diagnostic steps. Our earlier survey also presents the perspectives of a variety of clinical specialists in more detail, including surgery, internal medicine, anesthesia, and general radiologists. To our knowledge, no other data is currently available on emergency radiologists’ perspectives concerning the role of CECT in patients with sepsis.

Another important issue is that the use of ionizing radiation should be restricted to avoid exposing patients unnecessarily [22]. However, in conditions with a high mortality rate, such as sepsis, radiation exposure associated with repeat imaging appears to be justifiable. The survey results suggest that emergency radiologists are more likely to accept clinical reasoning in specific scenarios—such as in septic patients experiencing clinical deterioration—where additional imaging may be considered beneficial. Given the absence of data pertaining to the rationale behind individual responses within the survey, the validity of this interpretation remains speculative. Data from physicians’ perspectives regarding contrast administration is scarce and therefore clearly needed. Importantly, European Society of Urogenital Radiology (ESUR) guidelines provide guidance regarding the use of contrast agents in specific risk populations such as patients with kidney failure [23]. It remains uncertain how rigorously these guidelines are adhered to in clinical practice. Furthermore, while ESUR guidelines provide clear recommendations on the general safe use of contrast media, an interdisciplinary consensus specific to imaging patients with sepsis may help define shared principles for balancing diagnostic benefit with risks such as contrast exposure, radiation, and patient transport.

This study has a few limitations. Firstly, the data from this survey among ESER radiologists was compared with a previous survey conducted in a large European university medical center and published in part before [16, 17, 21]. Data from the tertiary care hospital were stratified into a group of general radiologists and clinicians to provide a reference for comparison with the perspectives of ESER radiologists. As the results presented here thus derived from two surveys conducted at two different time-points (and in different countries), general conclusions should be drawn with caution. While radiologists specializing in general radiology at a large European university medical center may possess proficiency in emergency imaging, their expertise in this field is often considered to be less extensive. Importantly, data on the perspectives of ESER radiologists were obtained in a multi-center setting and compared with the findings obtained in a single tertiary center, as described. In addition, due to data protection regulations and the anonymous nature of the surveys, we cannot fully exclude potential overlap between respondents of the previous survey and the current ESER cohort. Although participants were clearly instructed to complete the questionnaire only once and the questionnaires were nearly identical, duplicate participation cannot be entirely ruled out. Secondly, a further distinction emerges with respect to the extent of professional expertise among the physician populations constituting both surveys, i.e., ESER radiologists are more experienced, potentially impacting the results. Therefore, we provided supplementary data to account for professional experience and exclude major bias. Thirdly, it is well known that voluntary survey participation leads to a self-selection bias. This is a limitation as not all ESER radiologists are represented in the analysis. The latter limitation applies equally to both datasets analyzed in this paper. Lastly, ESER membership is voluntary itself and may not represent a comparable level of knowledge in how to perform CECT in patients with sepsis.

The results of this survey indicate a high degree of consensus on the importance of promptly performing CECT to avoid delays in the diagnostic and further management of patients with sepsis. At the same time, differences between emergency radiologists and other specialties highlight the need to better align interdisciplinary workflows. These findings provide a foundation for future prospective studies and may contribute to more targeted and efficient diagnostic pathways in sepsis care.

## Supplementary information

ELECTRONIC SUPPLEMENTARY MATERIAL

## Abbreviations

CECT: Contrast-enhanced CT
ESER: European Society of Emergency Radiology
STROBE: Strengthening the reporting of observational studies in epidemiology
