# Supplementary material for: Contrast-enhanced CT in sepsis: insights from a European Emergency Radiology survey
Source: Eur Radiol. 2026 Jan 26;36(7):5987–95. doi: 10.1007/s00330-025-12256-y (PMC13282361; doi:10.1007/s00330-025-12256-y)
Supplement: Supplementary file 1 — ELECTRONIC SUPPLEMENTARY MATERIAL [file 330_2025_12256_MOESM1_ESM.pdf]

**Contrast-enhanced CT in Sepsis: Insights from a European Emergency Radiology Survey**
**ELECTRONIC SUPPLEMENTARY MATERIAL**

# ***Survey among European radiologists on the use of computed tomography in patients with sepsis***

## - Questionnaire -

## **START** (Information text + consent; request consent to participate prior to participation; some details on participants requested and collected before the actual questionnaire begins)

→ E-mail including brief information about the study:

Dear Colleagues,

We would like to ask you to participate in our study "Survey among European radiologists on the use of computed tomography for focus identification in patients with sepsis". This is a digital questionnaire provided by a study team of the Department of Radiology at Charité - Universitätsmedizin Berlin (Campus CCM).
Our study aims at investigating the role of computed tomography (CT) in the detection of septic foci in the eyes of doctors. Your views will help us to derive consequences for optimized patient care.
Completing the questionnaire takes about 10 minutes. The link sent with this e-mail is active for a period of 30 days [planned period; may vary if necessary]. Your data are collected anonymously and cannot be attributed to you. The data collected are used exclusively for scientific purposes. Participation in the study is voluntary and you can stop the survey at any time without giving a reason.
Please feel free to contact the Study Team if you have any further questions about our study. To participate in the study, please click on the following link:
LIN K: ____________________________________________

Thank you for your willingness to participate.

Kind regards.

Maria Isabel Opper Hernando, Professor Dr. Marc Dewey, and Dr. Julian Pohlan for the Study Team

Contact details of the Study Team: Dr. Julian Pohlan, julian.pohlan@charite.de, Luisenstr. 7, 10117, Berlin

Principal Investigator: Dr. Julian Pohlan

*Note: There is a free text passage in the survey. Please avoid providing any details that might reveal your identity. An example would be mentioning the clinic/hospital where you mainly work.*

## “Page 0”

### Information for participants and consent to participate in a medical research project

Information on the processing of the collected data:
For research purposes, we collect the following details on participants who complete the questionnaire: radiologist in training vs. board-certified radiologist, your professional experience in years since you were licensed to practice as a doctor, your predominant field of activity, and your professional opinion on the use of computed tomography (CT) in patients with sepsis (*including the following survey data: imaging with examination requirements and findings of the CT examination, suggestions for improvement of diagnostic procedures and interventional care, administration of contrast media*). As part of the study, we do not collect any sensitive personal data as defined in Art. 9 (1) of the General Data Protection Regulation (GDPR). LimeSurvey GmbH stores the information automatically sent by your browser when you access the website in a temporary so-called log file. This is based on Art. 6, paragraph 1, p. 1 lit. f GDPR, the legal basis for data processing in the setting of our survey. These data include, among other things, an anonymized (= abbreviated) IP address of the requesting computer, the time stamp of the access, the name and URL of the retrieved file, and other details on the browser and the operating system. These data will be deleted automatically and will not be used to re-identify participants. We ensure that we only cooperate with partners (order processing pursuant to Art. 28 GDPR) who take appropriate technical and organizational measures to protect your data. You can find out more about the processor, LimeSurvey GmbH, on the following page: https://www.limesurvey.org/de/.

Technical-organizational measures/standard:
The anonymous declaration of consent is stored electronically on *Charité’s internal servers (S-drive of Charité Radiology, CCM)*. Anonymous data collection precludes re-identification of your person.

Responsibility:
The Department of Radiology is responsible for processing the research data and is solely responsible for analyzing the research data. LimeSurvey GmbH is not a cooperation partner (with jointly responsible persons pursuant to Art. 26 GDPR), but a processor (Art. 28 GDPR). If you have any questions about the study, please contact the data processing officer (test center/principal investigator):

Dr. med. Julian Pohlan, MD
Berlin Institute of Health
Charité – Universitätsmedizin Berlin
Department of Radiology
Campus Charité Mitte, Charitéplatz 1, 10117 Berlin
Telephone: +49 30 450 627 006
E-Mail: julian.pohlan@charite.de

Publication:
We intend to publish the results of the study in a form that does not allow us to determine your identity.

Storage/Deletion:
To comply with the rules of good scientific practice, we are obligated to provide proof that the published data are based on original data for a retention period of 10 years. For this purpose, we store the collected data in electronic form on *Charité's internal servers (S-drive of Charité Radiology, CCM)*. As soon as the retention period has expired, the data are automatically deleted.

Description of the rights of participants:
Since this is an anonymous survey that does not allow conclusions to be drawn about a natural person, the assertion of the corresponding data subject's rights is restricted in this study (according to the GDPR). In detail, as follows:

The right

- to revoke consent. You can revoke your consent at any time. Please note that the lawfulness of the processing of your data carried out up to that time is not affected, Art. 7 paragraph 3 GDPR. This means that the data that have already been included in scientific evaluations are not affected by the revocation;
- to information about all data processed and stored about your person as well as the recipients to whom data are or have been passed on, Art. 15 GDPR;
- to rectification of inaccurate personal data, Art. 16 GDPR;
- to object to the further processing of your personal data, which is carried out without your consent due to public interest or to safeguard the legitimate interests of the controller. The objection to further processing must be justified so that it becomes clear that special circumstances justified in your person outweigh the aforementioned interest in further processing, Art. 21 GDPR;
- to erasure provided that certain reasons exist. This is particularly the case in the event of unlawful processing or if the data are no longer necessary for the purpose for which they were collected or processed, you withdraw your consent and there is no other legal basis for the data processing or instead of the aforementioned objection pursuant to Art. 21 GDPR under the conditions stated therein. If the deletion would destroy the objectives of a research project carried out in the scientific interest or make it significantly more difficult, there is no right to deletion, Art. 17 (3) GDPR;
- to restrict the processing of your personal data, in particular, if the processing is unlawful and you request the restriction instead of deletion (see there) or as long as it is disputed whether the processing of personal data is lawful, Art. 18 GDPR.

Your general right to complain to the competent supervisory authority pursuant to Art. 77 GDPR is not affected by the above restrictions.

If you believe that data processing is unlawful, you have the option of lodging a complaint with the supervisory authority responsible for Charité – Universitätsmedizin Berlin:
Berlin Commissioner for Data Protection and Freedom of Information
Friedrichstraße 219
10969 Berlin, Germany
Telephone: +49 30 13889-0
Fax: +49 30 2155050
E-Mail: mailbox@datenschutz-berlin.de

If you have any queries regarding data processing and compliance with data protection requirements, you can also contact the data protection officer of Charité - Universitätsmedizin Berlin:
Data Protection Officer of Charité - Universitätsmedizin Berlin
Charitéplatz 1
10117 Berlin, Germany
Telephone: +49 30 450 580016
E-Mail: datenschutzbeauftragte@charite.de

## Text of consent *(Opening after clicking on the sent link)*

I am aware that in this study no personal, particularly sensitive information about my health, ethnic origin, etc. is to be processed. The processing of the data occurs in accordance with Art. 6 para. 1 bed. a, and Art. 9 paragraph 2 beds. a of the General Data Protection Regulation (GDPR) and requires the submission of the following Declaration of Consent:

In the written information provided, I was informed in detail and comprehensibly that the data collected in the study are collected for the purposes described in the information sheet and stored and evaluated in an anonymous form. I agree that the study results can be published anonymously and that the records may be deposited in medical journals.

I am aware that, due to the anonymous nature of the survey, it is not possible to trace my person and therefore the rights of data subjects to access, correct, and delete my data are restricted. My data cannot be re-identified. I can also complain to a data protection authority.

I have had enough time to decide to participate in the study and know that my participation is voluntary. I have been informed that I can stop processing the questionnaire at any time without giving any reason. I hereby declare my voluntary participation in this study.

- ☐ I consent to the data processing under the stated conditions and would like to participate in the survey.

PLEASE CLICK HERE
TO PARTICIPATE
IN THE SURVEY

If you do not agree to the data processing as described above, please close the survey window.
AFTER CLICKING THE "CONTINUE" BUTTON:

## 1.

Is this the first time that you complete the questionnaire?
If you wish to participate and have not completed the questionnaire before, please tick the "YES" box to start the survey. If you have already completed the questionnaire, we kindly ask you to tick the "NO" box and not participate again.

- ☐ YES
- ☐ NO

PLEASE CLICK HERE TO CONTINUE

## 2. Demographics/Study population

**A) Professional experience in the field of radiology**

- ☐ Assistant physician (radiologist in training)
- ☐ Board-certified (Specialist/Attending) physician
- ☐ Senior or Chief physician
    - ○ Professional experience since license to practice as a doctor
      [This opens with all tick options.]
        - ○ < 3 years
        - ○ > 3 - ≤ 7 years
        - ○ > 7 - ≤ 11 years
        - ○ > 11 - ≤ 20 years
        - ○ > 20 years

**B) Workplace/ Functional field / Primary line of work** (>50% of working hours)

- ☐ Radiology department at a hospital
- ☐ Radiology department as part of the emergency department
- ☐ Outpatient radiology facility
- ☐ Other: _________

**C) Primarily used modality** (>50% of working hours)

- ☐ Computed tomography (CT)

☐ Ultrasonography (USG)
☐ Magnetic resonance imaging (MRI)
☐ Radiography (x-ray)
☐ Mammography
☐ Other: ____________

D) **<u>Specialties that request CT for patients with sepsis in my workplace:</u>**
Please rank the three specialties by order of importance in requesting CT scans for focus search in patients with sepsis, giving your personal opinion *(1 = most important to 3 = least important)*

☐ Emergency department
☐ General ward
☐ Intensive care unit (ICU)

E) In which country do you practice medicine? [Free text field]

F) **<u>I perform CT-guided interventions such as drainages and punctures in patients with sepsis.</u>**
☐ Yes
☐ No

G) **<u>Only mark what is applicable to you:</u>**
☐ I am not involved in the management (diagnosis and/or treatment) of septic patients.
☐ I am involved in the management (diagnosis and/or treatment) of septic patients.

## 3. First Subset of Questions: CT indication in patients with sepsis

Please indicate your personal preferences and views on the management of patients with sepsis below.

| *Please select one answer each.* | | | | |
|---|---|---|---|---|
| The following clinical criteria are arguments for a CT request in patients with suspected sepsis: | *Strongly disagree* | *Somewhat disagree* | *Somewhat agree* | *Strongly agree* |
| SOFA[*a] score increased by ≥ 2 points | | | | |
| qSOFA score criteria ≥ 2 | | | | |
| Fever or hypothermia | | | | |
| SIRS criteria[*b] ≥2 | | | | |
| Respiratory rate ≥ 22/min | | | | |
| Postoperative patient (surgery in the last 7 days) | | | | |
| Increasing catecholamine demand | | | | |
| Signs of reduced vigilance/ altered mental status | | | | |
| Immunosuppression (due to medication and/or pre-existing illness) | | | | |
| SBP < 100 mmHg or MAD < 65 – 70 mmHg | | | | |
| Elderly patient (>65 years old) | | | | |
| The following additional parameter is of particular relevance for the indication of CT... | *Strongly disagree* | *Somewhat disagree* | *Somewhat agree* | *Strongly agree* |
| Elevated PCT | | | | |
| Elevated CRP | | | | |
| Leukocytosis or leukopenia | | | | |

| | | | | |
|---|---|---|---|---|
| Elevated IL-6 | | | | |
| Elevated lactate levels | | | | |
| Sonographically suspected infection focus | | | | |
| Pathological chest x-ray | | | | |
| SARS-CoV-2 detected | | | | |
| Positive blood culture | | | | |

*Sequential organ failure assessment (SOFA), Systemic inflammatory response syndrome (SIRS), Systolic blood pressure (SBP), Mean arterial pressure (MAD), Procalcitonin (PCT), C-reactive protein (CRP), Interleukin 6 (IL-6),*
*[a] *SOFA score criteria:* lung function, kidney function, liver function, circulatory function, CNS, blood count
*[b] SIRS criteria: body temperature, respiratory rate, heart rate, blood count

**H)** What is your preferred imaging modality when searching for a septic focus in patients with sepsis without a clinically suspected focus?
(Arrange the elements in the order of your preference (preferred at the top). The elements can be moved with the mouse. Double click moves an element to the other list.)

- ☐ CT
- ☐ MRI
- ☐ USG (Ultrasonography)
- ☐ X-ray

**I)** Please prioritize the organ regions, as your preferred protocol for focus search in septic patients (Rank 1 to 5).
(Arrange the elements in the right list (highest rating at the top). The elements can be moved with the mouse. Double click moves an element to the other list.)

SELECTION:

- Chest or Abdomen according to clinical assessment
- Chest+Abdomen+Pelvis
- Head+Chest+Abdomen+Pelvis
- Head+Neck+Chest+Abdomen+Pelvis
- Head+Neck+Chest+Abdomen+Pelvis+Legs

## 4. Second Subset of Questions: Use of computed tomography

| *Please select one answer each.* | | | | |
|---|---|---|---|---|
| If the initial CT fails to detect a focus, … | *Strongly disagree* | *Somewhat disagree* | *Somewhat agree* | *Strongly agree* |
| I would rely on further diagnostic tests such as microbiology. | | | | |
| I would opt for a repeat CT scan in case of clinical deterioration. | | | | |
| and the patient has improved clinically, I would request a follow-up CT after 1 week. | | | | |
| I would use alternative imaging (sonography, MRI, PET-CT, etc.) | | | | |
| and the patient is clinically unaltered, I would like to conduct a repeat CT scan. | | | | |
| [IF ABOVE “Somewhat agree” OR “*Strongly agree*”] If you opt for a repeat CT scan after an initial CT is focus-negative: what do you consider the best time window? | within 24h after the CT scan without detected focus | within 3 days | within 4 to 7 days | After 7 days |
| What do you consider the best time window for a CT scan after the diagnosis or suspected diagnosis of sepsis in a patient without a clinically suspected focus. | <1h | ≥1-6h | ≥6-12h | ≥12-24h |
| I see the greatest benefit of CT… | *Strongly disagree* | *Somewhat disagree* | *Somewhat agree* | *Strongly agree* |
| in confirming the clinically suspected focus. | | | | |
| in the ability to detect a previously unknown focus. | | | | |
| in the modification of anti-infectious therapy. | | | | |
| in planning interventions (e.g., drainage, puncture) and/or surgeries. | | | | |

| | | | | |
|---|---|---|---|---|
| in exclusion diagnosis. | | | | |
| State your preference regarding the time window after a CT scan in which possible CT-guided interventions (drainage, puncture, etc.) should take place. | <1h | ≥1-6h | ≥6-12h | ≥12-24h |
| Which clinical scenario applies to you in the management of patients with sepsis? | *Strongly disagree* | *Somewhat disagree* | *Somewhat agree* | *Strongly agree* |
| I perform a CT scan for sepsis because the benefits outweigh the side effects (radiation exposure, contrast agent risks). | | | | |
| Due to the radiation exposure, I do without a CT scan in patients with sepsis if possible. | | | | |
| I prefer to conduct unenhanced CT scans because I am concerned about contrast agent side effects. | | | | |
| Before I perform a CT scan, there should be an ultrasound examination. The subsequent CT serves to confirm the findings. | | | | |
| In patients with a confirmed SARS-CoV-2 infection, I definitely perform a CT scan. | | | | |
| Before I perform a CT scan, there should be an ultrasound examination. The subsequent CT serves to find additional or new foci. | | | | |

*Magnetic resonance imaging (MRI), Positron emissions tomography - Computed tomography (PET-CT)*

## 5. Fourth Subset of Questions: Contrast agent use

Please indicate your preferences regarding intravenous contrast agent use in CT examinations of patients with sepsis. Please report your experience in clinical practice. Note: Please do not simply close the window tab if you do not wish to answer the sub-items listed here. The questionnaire can also be analyzed without completing this group of questions. Please click the "Submit" button if you wish to stop here.

**A)** Indication for intravenous contrast-enhanced CT in individuals with sepsis.

| *Please select one answer each.* | | | | |
|---|---|---|---|---|
| | Definitely without contrast agent | Preferably without contrast agent | Preferably with contrast agent | Definitely with contrast agent |
| Focus search in abdominal CT | | | | |
| Focus search in chest CT | | | | |
| Focus search in CT of the Chest+Abdomen+Pelvis | | | | |
| Focus search in cranial CT (head CT) | | | | |
| Focus search in neck CT | | | | |
| Focus search in teeth CT | | | | |
| Focus search in paranasal sinuses CT | | | | |
| Focus search in legs CT | | | | |

**B)** Contraindications to the use of intravenous contrast agent in the context of focus search

Imagine you have a patient with sepsis and additionally one of the complications listed in the table below. Indicate how you would decide on the administration of contraceptives in the respective situation in everyday clinical practice.

| *Please select one answer each.* | | | | |
|---|---|---|---|---|
| | Absolute contraindication to contrast agent | Relative contraindication to contrast agent | CT with contrast agent possible after appropriate preparation* | No contraindication to contrast agent |
| latent hyperthyroidism (TSH reduced, normal fT3) | | | | |
| clinical presentation with ileus | | | | |
| mild acute adverse reaction after previous contrast agent administration | | | | |
| manifest hyperthyroidism (TSH reduced/fT3 increased) | | | | |
| Creatinine >2mg/dl or eGFR <30ml/min | | | | |
| moderate acute adverse reaction after previous contrast agent administration | | | | |
| End-stage renal failure / hemodialysis | | | | |
| severe acute adverse reaction after previous contrast agent administration | | | | |
| Acute heart failure NYHA stage IV | | | | |

* Prophylaxis (including hydration and/or medication) or lower dose of contrast agent Thyroid-stimulating hormone (TSH), free triiodothyronine (fT3), estimated glomerular filtration rate (eGFR), New York Heart Association (NYHA) ACUTE ADVERSE REACTIONS mild acute adverse reaction after previous contrast agent administration: mild urticaria, mild itching, erythema; nausea/mild vomiting, warmth/chills, anxiety, vasovagal reaction which resolves spontaneously moderate acute adverse reaction after previous contrast agent administration: marked urticaria, mild bronchospasm, facial/laryngeal edema; vasovagal reaction severe acute adverse reaction after previous contrast agent administration: hypotensive shock, respiratory arrest, cardiac arrest; arrythmia, convulsion

SEND/SUBMIT (DATA)

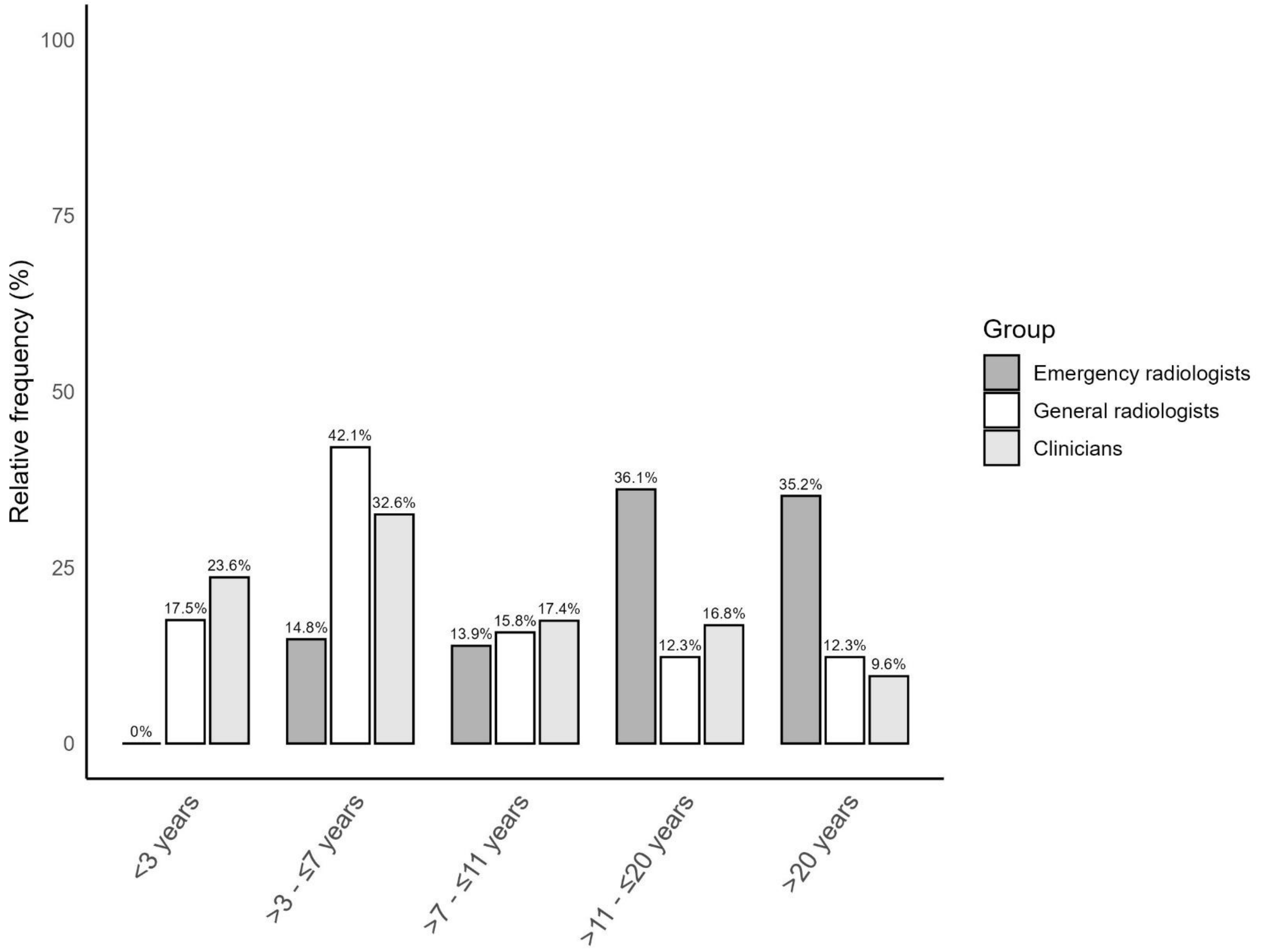


**Figure S1**

Professional experience in years of work experience

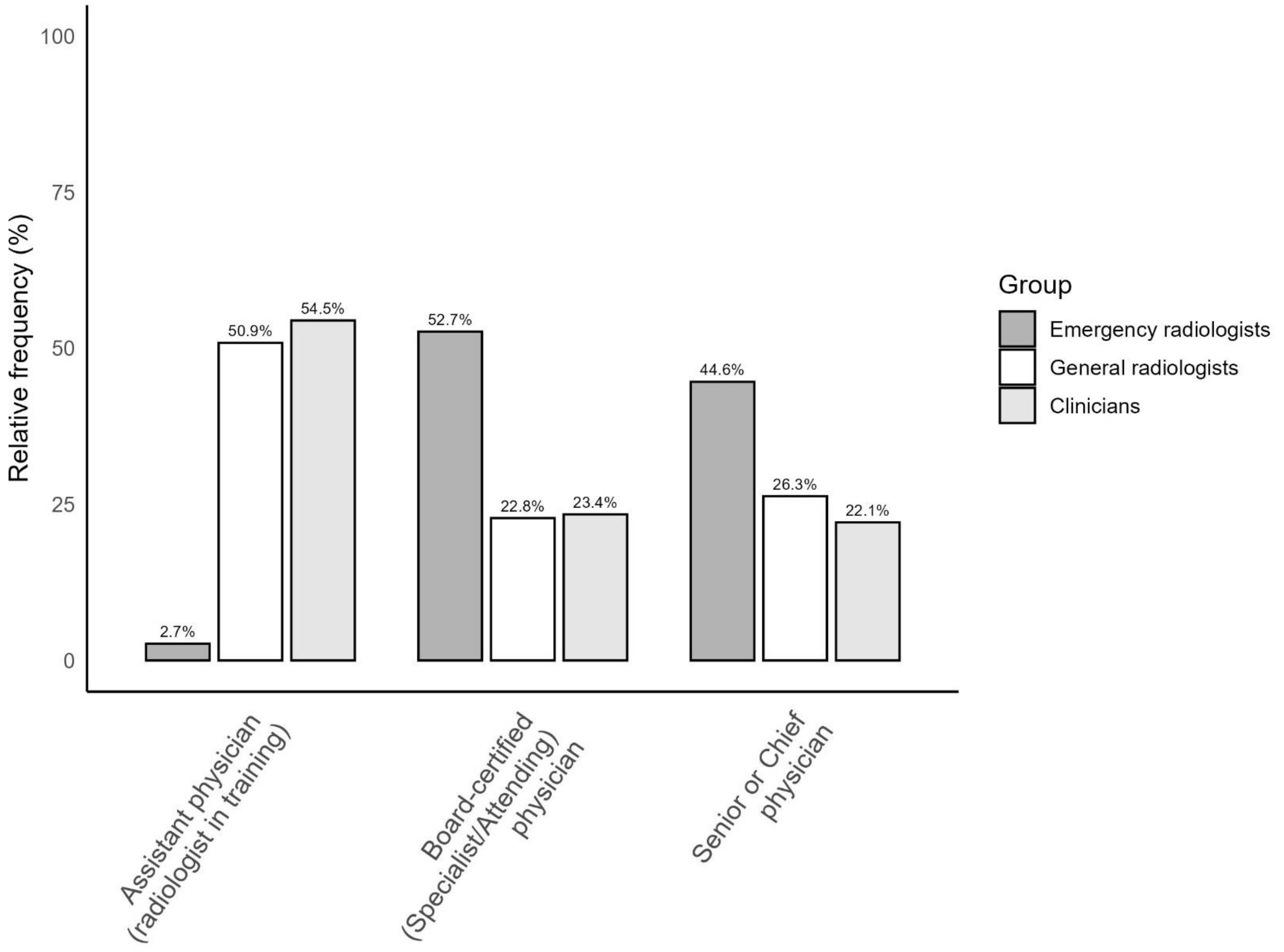


**Figure S2** Professional experience of survey participants with regard to board certification

Eur Radiol (2025) Stahl AC, Rubarth K, Hernando MIO, et al.

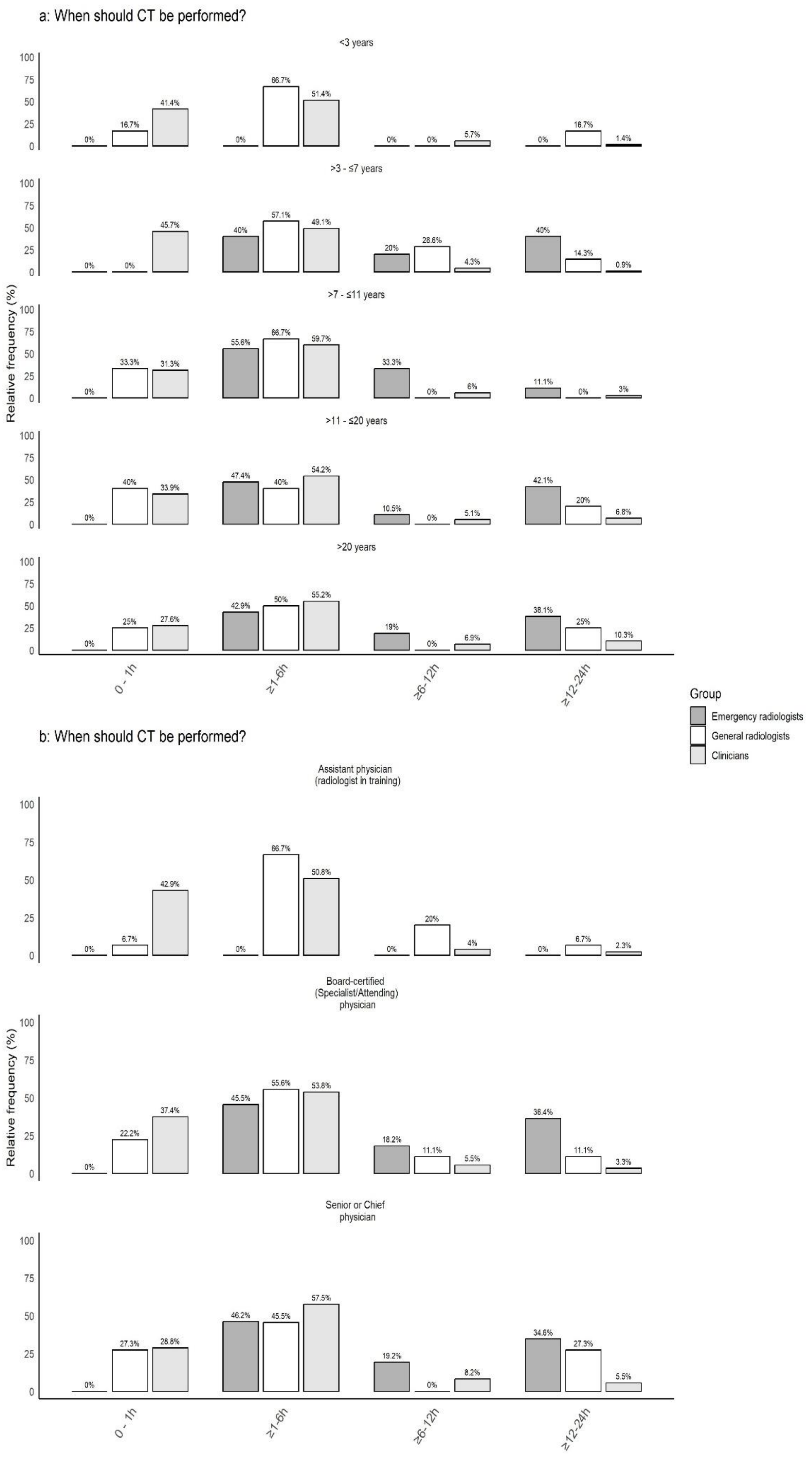


## Figure S3

Stratified analysis according to professional experience in years of experience (a) and board-certification (b) with a focus on optimal timing of CT

## Table S1 Preferred window of CECT timing in sepsis*

| *Time to CECT* | *Professional group* | | | Total |
|---|---|---|---|---|
| | Emergency radiologists | General radiologists | Clinicians | |
| 0 - 1h | 0<br>0% | 6<br>17.1% | 131<br>38.4% | 137<br>31.5% |
| ≥1-6h | 27<br>45.8% | 20<br>57.1% | 181<br>53.1% | 228<br>52.4% |
| ≥6-12h | 11<br>18.6% | 411.4% | 18<br>5.3% | 33<br>7.6% |
| ≥12-24h | 21<br>35.6% | 5<br>14.3% | 11<br>3.2% | 37<br>8.5% |
| Total | 59<br>100% | 35<br>100% | 341<br>100% | 435<br>100% |

*CECT* contrast-enhanced CT

*χ2=102.765; df=6; Cramer's V=0.344; Fisher's p=0.000

**Table S2 Should CECT be repeated in patients with clinical deterioration without focus identification in prior CT?***

| *Repeat CT* | *Professional group* | | | Total |
|---|---|---|---|---|
| | Emergency radiologists | General radiologists | Clinicians | |
| Strongly agree | 24<br>35.3% | 3<br>8.6% | 10<br>2.9% | 37<br>8.3% |
| Somewhat agree | 29<br>42.6% | 13<br>37.1% | 87<br>25.5% | 129<br>29.1% |
| Somewhat disagree | 10<br>14.7% | 10<br>28.6% | 191<br>56% | 211<br>47.5% |
| Strongly disagree | 5<br>7.4% | 9<br>25.7% | 53<br>15.5% | 67<br>15.1% |
| Total | 68<br>100% | 35<br>100% | 341<br>100% | 444<br>100% |

*CECT* contrast-enhanced CT

*$\chi 2$=106.424; df=6; Cramer's V=0.346; Fisher's p=0.000

**Table S3 Contraindications for CECT in patients with latent (a) and manifest (b) hyperthyroidism**

**a***

| *Latent hyperthyroidism* | *Professional group* | | | Total |
|---|---|---|---|---|
| | Emergency radiologists | General radiologists | Clinicians | |
| Absolute contraindication to contrast agent | 0<br>0% | 1<br>2.9% | 1<br>0.3% | 2<br>0.5% |
| Relative contraindication to contrast agent | 19<br>29.2% | 1<br>2.9% | 46<br>13.8% | 66<br>15.3% |
| CECT after appropriate preparation | 22<br>33.8% | 29<br>85.3% | 221<br>66.4% | 272<br>63% |
| No contraindication to contrast agent | 24<br>36.9% | 3<br>8.8% | 65<br>19.5% | 92<br>21.3% |
| Total | 65<br>100% | 34<br>100% | 333<br>100% | 432<br>100% |

b**

| *Manifest hyperthyroidism* | *Professional group* | | | Total |
|---|---|---|---|---|
| | Emergency radiologists | General radiologists | Clinicians | |
| Absolute contraindication to contrast agent | 9<br>13.8% | 1<br>5% | 3<br>1% | 13<br>3.4% |
| Relative contraindication to contrast agent | 24<br>36.9% | 10<br>50% | 99<br>32.8% | 133<br>34.4% |
| CECT after appropriate preparation | 21<br>32.3% | 7<br>35% | 193<br>63.9% | 221<br>57.1% |
| No contraindication to contrast agent | 11<br>16.9% | 2<br>10% | 7<br>2.3% | 20<br>5.2% |
| Total | 65<br>100% | 20<br>100% | 302<br>100% | 387<br>100% |

*CECT* contrast-enhanced CT

*$\chi 2$=39.627; df=6; Cramer's V=0.214; Fisher's p=0.000

**$\chi 2$=62.427; df=6; Cramer's V=0.284; Fisher's p=0.000

**Table S4 Contraindications for CECT in patients with prior acute adverse reactions divided into mild reactions (a) and severe reactions (b)**

**a***

| *Mild acute adverse reaction* | *Professional group* | | | Total |
|---|---|---|---|---|
| | Emergency radiologists | General radiologists | Clinicians | |
| Absolute contraindication to contrast agent | 1<br>1.5% | 1<br>2.9% | 10<br>3% | 12<br>2.8% |
| Relative contraindication to contrast agent | 13<br>20% | 2<br>5.9% | 70<br>21% | 85<br>19.6% |
| CECT after appropriate preparation | 43<br>66.2% | 23<br>67.6% | 233<br>69.8% | 299<br>69.1% |
| No contraindication to contrast agent | 8<br>12.3% | 8<br>23.5% | 21<br>6.3% | 37<br>8.5% |
| Total | 65<br>100% | 34<br>100% | 334<br>100% | 433<br>100% |

b**

| *Severe acute adverse reaction* | *Professional group* | | | Total |
|---|---|---|---|---|
| | Emergency radiologists | General radiologists | Clinicians | |
| Absolute contraindication to contrast agent | 47<br>71.2% | 14<br>41.2% | 144<br>43.2% | 205<br>47.3% |
| Relative contraindication to contrast agent | 10<br>15.2% | 11<br>32.4% | 102<br>30.6% | 123<br>28.4% |
| CECT after appropriate preparation | 7<br>10.6% | 7<br>20.6% | 85<br>25.5% | 99<br>22.9% |
| No contraindication to contrast agent | 2<br>3% | 2<br>5.9% | 2<br>0.6% | 6<br>1.4% |
| Total | 66<br>100% | 34<br>100% | 333<br>100% | 433<br>100% |

*CECT* contrast-enhanced CT

*$\chi 2$=16.114; df=6; Cramer's V=0.136; Fisher's p=0.015

**$\chi 2$=27.423; df=6; Cramer's V=0.178; Fisher's p=0.000

**Table S5 Contraindications for CECT in patients with impaired kidney function (a) or end-stage kidney failure (b)**

**a***

| *Impaired kidney function* | *Professional group* | | | Total |
|---|---|---|---|---|
| | Emergency radiologists | General radiologists | Clinicians | |
| Absolute contraindication to contrast agent | 12<br>18.5% | 1<br>2.9 % | 7<br>2.1% | 20<br>4.6% |
| Relative contraindication to contrast agent | 29<br>44.6% | 18<br>52.9% | 120<br>35.9% | 167<br>38.6% |
| CECT after appropriate preparation | 23<br>35.4% | 11<br>32.4% | 124<br>37.1% | 158<br>36.5% |
| No contraindication to contrast agent | 1<br>1.5% | 4<br>11.8% | 83<br>24.9% | 88<br>20.3% |
| Total | 65<br>100% | 34<br>100% | 334<br>100% | 433<br>100% |

b**

| *End-stage renal failure* | *Professional group* | | | Total |
|---|---|---|---|---|
| | Emergency radiologists | General radiologists | Clinicians | |
| Absolute contraindication to contrast agent | 4<br>6.2% | 1<br>2.9% | 18<br>5.4% | 23<br>5.3% |
| Relative contraindication to contrast agent | 16<br>24.6% | 4<br>11.8% | 65<br>19.5% | 85<br>19.6% |
| CECT after appropriate preparation | 17<br>26.2% | 6<br>17.6% | 58<br>17.4% | 81<br>18.7% |
| No contraindication to contrast agent | 28<br>43.1% | 23<br>67.6% | 193<br>57.8% | 244<br>56.4% |
| Total | 65<br>100% | 34<br>100% | 334<br>100% | 433<br>100% |

*CECT* contrast-enhanced CT

*$\chi 2$=50.915; df=6; Cramer's V=0.242; Fisher's p=0.000

**$\chi 2$=7.542; df=6; Cramer's V=0.093; Fisher's p=0.286
